# Analysis of the sensitization activity of *Moringa oleifera* leaves protein

**DOI:** 10.3389/fnut.2024.1509343

**Published:** 2025-01-22

**Authors:** Juan Lu, Xiaoxue Liu, Wenjie Li, Chuyu Xi, Dan Feng, Shuang Song

**Affiliations:** ^1^Yunnan Agricultural University, Kunming, China; ^2^Yunnan Provincial High Court Characteristic Agricultural Industry Research Institute, Kunming, China; ^3^College of Food Science and Technology, Yunnan Agricultural University, Kunming, China

**Keywords:** *Moringa oleifera* leaves protein, allergy, allergy-related activities, hemagglutination, hemolytic activity

## Abstract

The determination of allergenic proteins in *Moringa oleifera* leaves, which is the main components of immune activity, has enabled the development of a more effective method for evaluating the activity of extracted *Moringa oleifera* leaves protein. In this study, the extraction process of *Moringa oleifera* leaves protein was optimized based on a single factor experiment. The hemagglutination-related properties of *Moringa oleifera* leaves protein, such as (thermal, acid–base) stability, sugar binding specificity, ion binding characteristics, and hemolytic activity, were detected. The optimal combination of extraction process was: extraction time of 6 h, material-liquid ratio of 1:8, and ammonium sulfate saturation of 60%. The extraction rate of moringa leaf protein under this condition was 14.37 mg/g. The molecular weight of moringa leaf protein was analyzed by SDS-PAGE, and the molecular weight was mainly concentrated around 23 kDa~70 kDa, with the highest content of 35 kDa (major allergen). The study of the hemagglutination characteristics of *Moringa oleifera* leaves protein revealed that the protein exhibited high stability at temperatures below 60°C, with complete loss of activity occurring at temperatures above 110°C for 20 min. The effect of different pH conditions on the hemagglutination capacity of *Moringa oleifera* leaves protein was readily discernible. The hemagglutination activity of *Moringa oleifera* leaves protein was 10^4^ in a pH value from 3.7 to 7.8, and the hemagglutination activity was completely lost at a pH value higher than 11.9. D(+) anhydrous glucose is the specific inhibitory sugar of *Moringa oleifera* leaves protein lectin. *Moringa oleifera* leaves protein exhibits hemolytic activity at a concentration of at least 20 mg/mL, and α-methyl-mannoside, galactoside, raffinose and Al^3+^ can inhibit the hemolysis of *Moringa oleifera* leaves protein. The present study identified the effects of different factors on the coagulation activity and hemolytic ability of *Moringa oleifera* leaves protein, thereby providing a theoretical basis for further purification and application of *Moringa oleifera* lectin. However, it should be noted that the results of the mixture have certain limitations, and further purification of lectin is needed to obtain more targeted research results.

## Graphical Abstract

The effects of different factors on the coagulation activity and hemolytic ability of *Moringa oleifera* leaves protein. 
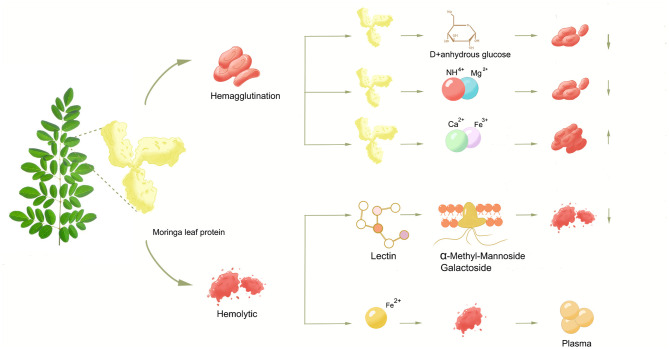

## 1 Introduction

*Moringa oleifera (M. oleifera)*, is commonly known as drumstick tree. This crop grows rapidly, has strong adaptability and is widely planted in tropical and subtropical areas of Asia and Africa (1, 2). *M. oleifera* leaves were approved as a new resource food in 2012 by China National Food Industry Association due to their rich nutritional value (3) and have been widely consumed as a special food in many regions. *M. oleifera* is a versatile tree species. Its roots and bark can be used as a traditional medicinal materials, fresh leaves rich nutrition, can be used as edible vegetables, seeds are rich in fat, which can be used to press oil (4). *M. oleifera* is a rich source of vitamin A, vitamin B, vitamin C, vitamin E, as well as other nutrients, including potassium, calcium, iron, and various minerals (5). In addition, it is rich in essential amino acids and trace elements required by the human body, and its nutritional value is comparable to that of Spirulina, which enjoys the reputation of “the micro-treasure house of human nutrition” (5, 6). People in many countries and regions of the world have the habit of eating *M. oleifera* to promote health care. For example, in India, *M. oleifera* leaves are said to have the effect of lowering cholesterol and are used to treat heart disease and obesity (7, 8).

*M. oleifera* leaves are sources of high-quality plant protein. Satish et al. (9) also found the hydrolysis activity of casein in *M. oleifera* leaves. *M. oleifera* is an important source of protease inhibitors. Bijina et al. (10) extracted and purified a protease with a molecular weight of 51 kDa from *M. oleifera* leaves. The protease exhibited optimal activity at pH 8 and 37°C, with the highest casein hydrolysis activity observed under these conditions. The identified proteins are mainly involved in energy exchange and metabolism and in carbohydrate and protein metabolism. Among the identified proteins, proteases are mainly involved in the curd activity of milk. Pusztai et al. (11) used *M. oleifera* protein and alum for the treatment of turgor water and found that *M. oleifera* protein is better than alum in removing turgor water and *E. coli*.

*M. oleifera* leaves are rich in high-quality protein, but in the process of *M. oleifera* promotion, it is found that some people will have rash, diarrhea, abdominal pain, dizziness, nausea, vomiting and other symptoms after eating *M. oleifera* leaves, especially fresh leaves, which is particularly obvious (7). These adverse symptoms are thought to be caused by disorder of immune allergic reaction (12). Xi et al. (13) and Zhang et al. (14) haved successfully sensitized BALB/c mice to *M. oleifera* leaves protein through oral administration. D'Auria et al. identified potential *M. oleifera* leaves allergens, including morintides and nsLTP, through proteomic analysis (15). Iddagoda et al. determined the molecular weights of the *M. oleifera* leaves allergens to be 14, 23, 35, 43, and 48 kDa using Western blotting with serum from patients with *M. oleifera* leaves allergy (16). Most plant lectins in digestive system can enzyme degradation resistance, resistance to degradation of lectins in combination with surface receptor in the digestive tract, swallowing and lectin receptors was within the cell, through the epithelial cells into the circulation of the blood, produce anticoagulant set IgE (allergic reaction) and IgG antibody (9–12). In this study, we hypothesized that the major allergenic protein in *M. oleifera* leaves protein is *M. oleifera* lectin, and studying the basic properties of lectins in *M. oleifera* leaves protein was the first step to confirm this hypothesis, so its properties were investigated. Hev b 6 (Hevein, a lectin-like protein and a major allergen of latex) was predicted as the most similar allergen to the Morintides mO1 and mO2 proteins (15). That will support our hypothesis that “the major allergenic protein in *M. oleifera* leaves protein is *Moringa oleifera* lectin”. The existing methods for the detection and analysis of lectins are mainly carried out by using the properties of lectins, including cell agglutination, sugar binding properties and protein properties. Plant lectins have the ability to agglutinate red blood cells from rabbits and humans, and the hemagglutination activity (agglutination potency) is linearly related to the amount of lectins (9–11). Use lectins, therefore, all natural or enzyme treatment of people or animals to the characteristics of red blood cells or red blood cell agglutinate method (blood clot) (Hemagglutination) to test the lectin. Lectins can also be detected on the basis of their characteristic of binding specifically to sugars, glycoproteins, or sugar-binding proteins, namely the sugar-complex method (11, 12). Based on the above analysis, it can be seen that the hemagglutination method has the advantages of simple operation, rapid reaction, obvious effect, and less influence by conditions. In this experiment, *M. oleifera* leaf protein (lectin) was detected by hemagglutination method, and the hemagglutination activity of lectin was used to measure the activity of lectin. In this study, the thermal stability, acid-base stability, ion binding properties, sugar binding specificity and hemolytic activity of *M. oleifera* leaves protein or lectin were studied to provide theoretical basis for the development and utilization of *M. oleifera* protein and its agglutination active substances.

## 2 Materials and methods

### 2.1 Materials and chemicals

Fresh *M. oleifera* leaves were obtained from Tianyou Technology Co. of Dehong State. Phosphate-buffered saline was purchased from Beijing Solarbio Technology Co. Ammonium sulfate and methyl alcohol were purchased from Beijing Chemical Reagent Factory, and a BCA (Bicinchoninic Acid) protein quantification kit was purchased from Beyotime. Dialysis bag (3,000 Da) were purchased from Shanghai Yuanye Biotechnology Co., Ltd., and two-color prestained markers were obtained from Yase Biotechnology Co., Ltd. Defibrinated rabbit blood from Beijing Solaibao Technology Co.

### 2.2 Extraction of *M. oleifera* leaves protein

The protein of *M. oleifera* leaves was extracted according to the improved operation method described by Feng and Wang (8) and Satish et al. (9). Fresh *M. oleifera* leaves were used, and phosphate buffer solution was added at a material-to-liquid ratio of 1:6. *M. oleifera* leaves were broken and homogenized, placed in a refrigerator at 4°C for extraction for 4 h, and stirred every 1 h. The residue was then filtered through gauze, and the filtrate was centrifuged at 1,200*g* for 20 min using a centrifuge to collect the supernatant. Subsequently, 472 g of ammonium sulfate crystals was added to each 1 L of sample solution and allowed to precipitate overnight, and the ammonium sulfate concentration was 70% to completely dissolve the ammonium sulfate crystals (15, 16). The samples were centrifuged again at 1,200*g* for 20 min, and the supernatant was discarded, leaving the precipitate. Subsequently, PBS (phosphate buffer saline) was added to dissolve the precipitate, and the precipitate was placed into the dialysis bag for dialysis for 2–4 days until the dialysis water became clear. The dialyzed *M. oleifera* leaves protein was then placed into a freeze-drying dish for vacuum freeze-drying to obtain *M. oleifera* leaves protein.

### 2.3 Single-factor test

Using the *M. oleifera* leaves protein extraction rate as the index to be investigated, various types of extract (normal saline, PBS, ultrapure water, Tris-HCl), different ammonium sulfate saturation levels (60, 70, 80%), different solid–liquid ratios (1:6, 1:8, 1:10), and various extraction times (6, 8, 10 h) were studied. The effects of these four factors on the extraction yield of *M. oleifera* leaves protein were assessed to determine the level of each factor in the optimal process.

### 2.4 Orthogonal experimental design

Through single-factor tests, the single-factor extraction conditions that yielded the best *M. oleifera* leaves protein yield were obtained. To optimize the protein extraction content, an orthogonal experimental design was selected as shown in Table 1.

**Table 1:** Table of factors and levels.

**Level**	**Factor**		
	**Extraction time (h)**	**Solid–liquid ratio**	**Ammonium sulfate concentration (%)**
1	6	1:6	60
2	8	1:8	70
3	10	1:10	80

### 2.5 Determination of the *M. oleifera* leaves protein concentration

According to the method described by Dai et al. (27) and Huang et al. (28), the absorbance value was measured at a wavelength of 595 nm, and bovine serum protein was used as the standard protein to obtain the standard curve, where Y is the protein concentration and X is the absorbance value. The protein extraction rate in solution was used as the reference to determine the best conditions for each process.

### 2.6 SDS–PAGE analysis

The experiment was performed according to the method described by Zenteno et al. (12) with a few modifications. The loading volume was calculated according to the *M. oleifera* leaves protein concentration. *M. oleifera* leaves protein and 5 μL of sample loading solution were added into a 0.2-mL centrifuge tube, and the sample was boiled and denatured for 10 min at 95°C. A 10% separation gel and 4% concentration gel were used. After preparation of the gel, the prepared *M. oleifera* leaves protein sample was added to the gel for electrophoresis. After electrophoresis, the gel blocks were removed, immersed in fixative solution for 30 min and then stained with Coomassie brilliant blue staining solution in a shaker. After staining, the dye was decolorized until the gel background was clean. WD-9403 gel rapid imager (Beijing Liuyi Biotechnology Co., Ltd) was used for photo analysis.

### 2.7 Determination of agglutination activity of *M. oleifera* leaves protein

#### 2.7.1 Determination of the hemagglutination titer

A 2% rabbit blood cell suspension was prepared as previously described (13). Using a total of 6 wells of type V blood clots, 25 μL of 0.9% saline was successively added to each well using a pipette gun. Twenty-five microliters of *M. oleifera* leaves protein was added to the first well of each row. Double dilution was performed (except in the last well of each row, which was used as a blank control), and 25 μL of 2% rabbit red blood cell suspension was then added to each well. The plate was shaken on a micro-oscillator for 1 min to ensure that the mixture was fully mixed. Hemagglutination was observed visually after 2 h at room temperature and denoted by 2^n^ (where n is the number of holes).

#### 2.7.2 Determination of the thermal stability of *M. oleifera* leaves protein

Lyophilized *M. oleifera* leaves protein was dissolved in PBS (pH 7.4, 0.01 mol/L) to obtain a solution with an initial concentration of 5 mg/mL. The solution was heated in a water bath, and the temperature was increased starting from 20°C and maintained constant for 5 min every 10°C. The *M. oleifera* leaves protein solution was removed, cooled at room temperature, and diluted in a 96-well “V” type hemagglutination plate. The hemagglutination activity was measured 2 h later.

#### 2.7.3 Determination of the sugar-binding specificity of *M. oleifera* protein

The test sugar was prepared in 0.5 mol/L mother liquor, 50 μL was added to well No. 1 of a 96-well “V” type hemagglutinin plate, and 50 μL of PBS buffer (pH 7.4, 0.01 mol/L) was added to wells No. 2–9. To each well,50 μL of *Moringa* leaves protein solution was added, and after 30 min at room temperature, 50 μL of red blood cell suspension was appended. The plate was allowed to stand for 2 h before the hemagglutination activity was measured. The following saccharides were used: α-methyl-D-glucopyranoside, maltose-hydrate, D-mannose, D(+) anhydrous glucose, D-raffinose pentahydrate, methyl-α-D-glucopyranoside, lactose, and methyl-β-D-galactoside.

#### 2.7.4 Determination of the ion-binding properties of *M. oleifera* leaves protein

*Moringa oleifera* leaves protein solution was prepared with 0.01 mol/L PBS buffer (pH 7.4) and set aside. Fifty microliters of 11ion solutions (0.1 mol/L) were added to each well of the 96-well “V” hemagglutination plate and then diluted with PBS buffer (pH 7.4, 0.01 mol/L). Subsequently, 50 μL of *Moringa oleifera* leaves protein solution was added to each well, and the hemagglutination activity was detected.

#### 2.7.5 Determination of the acid–base stability of *Moringa oleifera* leaves protein

*Moringa oleifera* leaves protein solution with an initial concentration of 5 mg/mL was prepared in a series of buffer systems with pH values from 3.7 to 13.0. The solution was left at room temperature for 2 h, and the hemagglutination activity under different pH conditions was measured after multiple dilution. The following buffers were used: NaCl-HAc buffer (0.1 mol/L) at pH 3.7–5.8, 0.1 mol/L NaH_2_PO_4_-Na_2_HPO_4_ buffer at pH 6.2–7.8, 0.1 mol/L Tris-HCl buffer at pH 8.3–9.1, 0.1 mol/L Na_2_CO_3_-NaHCO_3_ buffer at pH 9.5–10.8, 0.1 mol/L Na_2_HPO_4_-NaOH buffer at pH 11.2–11.9, and 0.1 mol/L KCl-NaOH buffer at pH 12.2–13.0.

### 2.8 Determination of the hemolytic activity of *M. oleifera* leaves protein

#### 2.8.1 Effect of *M. oleifera* leaves protein at different concentrations on hemolytic activity

The hemolytic activity was determined using the assay (19). Briefly, PBS-washed rabbit erythrocytes were prepared in 0.1 M PBS (pH 7.2, 1:9 v/v) containing 10 mM calcium chloride. Fifty microliters of various concentrations of *M. oleifera* leaves protein (5, 10, 20, and 40 mg/mL) was incubated with 950 μL of rabbit red blood cells (1:20 v/v) and centrifuged (2,000 rpm, 5 min). The absorbance of the supernatant (erythrocyte lysate) was read at 540 nm using a microplate reader. Erythrocytes incubated in PBS (pH 7.2) were used as a negative control, and 20% Triton X-100 was used as a positive control. The percentage of hemolysis was calculated using the following formula:


(1) $$\begin{array}{l} \text{a}=\text{Z}\left(\text{\%}\right)=\left(\text{At}-\text{Anc}\right)/\left(\text{Apc}-\text{Anc}\right)\times 100\text{\%} \end{array}$$

where a is the percentage of hemolysis, Z is the erythrocyte hemolysis rate, At is the absorbance of the test group, Anc is the absorbance of the negative control group, and Apc is the absorbance of the positive control group.

#### 2.8.2 Effect of sugar on the hemolytic activity of *M. oleifera* leaves protein

*M. oleifera* leaves protein (20 mg/mL) was incubated with 0.2 M maltose-hydrate, α-methyl-D-glucopyranoside, D-mannose, D(+) anhydrous glucose, D-lactate pentahydrate, lactose and methyl-β-D-galactoside for 2 h at 25°C and then with 10% freshly prepared rabbit red blood cell suspension (200 μL) at 30-min intervals with gentle shaking for 4 h. The samples were centrifuged (2,000 rpm, 5 min), and the absorbance values of red cell lysates were analyzed at 540 nm. To calculate the percentage of hemolysis, erythrocytes incubated in PBS (pH 7.2) were used as a negative control, and 20% Triton X-100 was used as a positive control.

#### 2.8.3 Effect of ions on the hemolytic activity of *M. oleifera* leaves protein

*M. oleifera* leaves protein (20 mg/mL) was incubated with MgCl_2_, KCl, FeCl_3_, CaCl_2_, NaCl, FeCl_2_, NH_4_Cl, BaCl_2_, and AlCl_3_ stock solutions for 2 h at 25°C and then with 10% freshly prepared rabbit red blood cell suspension (200 μL) with gentle shaking at 30-min intervals for 4 h. Samples were centrifuged (2,000 rpm, 5 min), and the absorbance values of red cell lysates were analyzed at 540 nm. To calculate the percentage of hemolysis, erythrocytes incubated with PBS (pH 7.2) were used as a negative control, and 20% Triton X-100 was used as a positive control.

### 2.9 Statistical analysis

All the data in this study are presented as the means from three or more independent experiments ± the standard deviations (SDs). The data were analyzed using GraphPad Prism software (7.0.0), and one-way analysis of variance (ANOVA) was used for comparisons among groups. ^*^P < 0.05 indicates a statistically significant difference.

## 3 Results

### 3.1 Single-factor experiments

#### 3.1.1 Effect of the extractant type on the protein extraction rate

**Figure 1:**
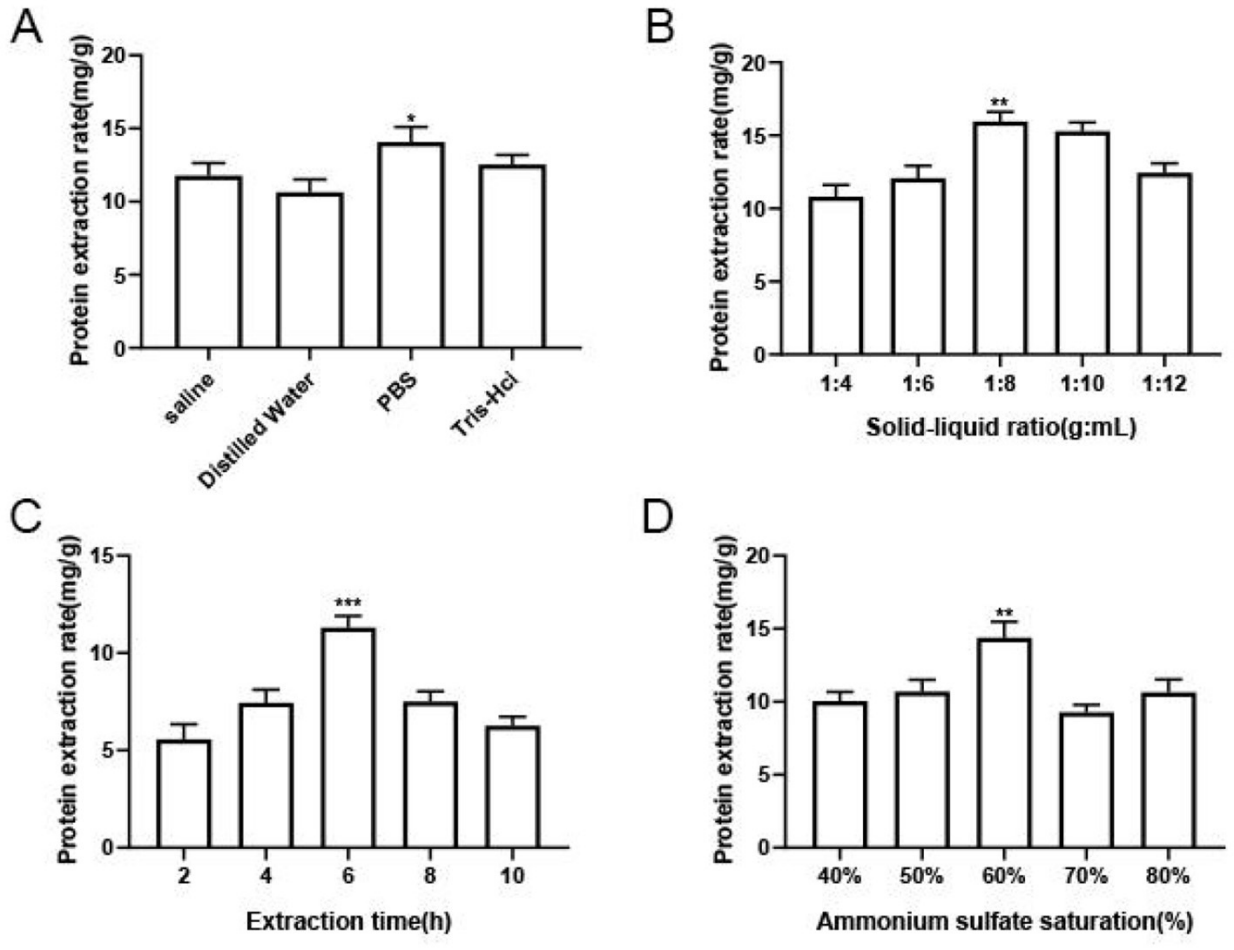
Results of the single-factor experiments. **(A)** Effect of the extractant type on the protein extraction rate. **(B)** Effect of the extraction time on the protein extraction rate. **(C)** Effect of the solid-to-liquid ratio on the extraction rate of *M. oleifera* leaves protein. **(D)** Effect of the ammonium sulfate saturation level on the extraction rate of *M. oleifera* leaves protein. The significance of the differences between groups is indicated as follows *P < 0.05, **P < 0.01, and ***P < 0.001.

With an extraction time of 6 h, a saturation level of ammonium sulfate equal to 60%, and a solid-to-liquid ratio of 1:8, the effect of the type of extract on the protein extraction rate of *M. oleifera* leaves is shown in Figure 1A. The protein extraction rates of the Tris-HCl and PBS groups were significantly higher than those of the other groups, and that of the PBS group was higher. This finding may be related to the strong buffering effect of PBS buffer, which can better maintain the structure and activity of the protein; this finding is consistent with the results reported (14). Therefore, PBS was selected as the optimal extract for *M. oleifera* leaves protein.

#### 3.1.2 Effect of the extraction time on the protein extraction rate

The effect of extraction time on the extraction rate of *M. oleifera* leaves protein was studied under the condition of 60% ammonium sulfate saturation and solid-to-liquid ratio of 1:8. As shown in the Figure 1B, an increase in the extraction time from 2 to 6 h increased the extraction rate of *M. oleifera* leaves protein from 5.57 to 11.3 mg/g. However, increasing the extraction time to 10 h decreased the protein extraction rate. This finding was probably observed because intracellular protein-degrading enzymes also are dissolved during the longer extraction time, and these protein-degrading enzymes degraded part of the dissolved proteins, thereby reducing the protein extraction rate (15). The optimal *M. oleifera* leaves protein extraction rate of 11.3 mg/g that was obtained with an extraction time of 6 h, which was 2.03 times that obtained with an extraction time of 2 h.

#### 3.1.3 effect of the solid-to-liquid ratio on the extraction rate of *M. oleifera* leaves protein

Next, the effect of solid-liquid ratio on the protein extraction yield of *M. oleifera* leaves was explored at an extraction time of 6 h and ammonium sulfate saturation of 60%.leaves. As shown in the Figure 1C, an increase in the liquid-to-solid ratio from 1:4 to 1:8 increased the protein extraction rate of *M. oleifera* leaves from 10.89 to 15.96 mg/g. This rapid enhance in the protein extraction rate may be due to an appropriate growth in the solvent volume to improve the contact area for *M. oleifera* and augment its dispersion, which enhances the diffusion effect and thus advance the extraction rate (16). However, with a further increase in the solid-to-liquid ratio to 1:12, the protein in plant cells becomes completely dissolved, and the amount of solvent has no effect on the protein extraction rate (15). The results showed that once the soluble protein concentration reached its maximum value, an increase in the solid-to-liquid ratio decreased the protein concentration. Therefore, the optimal solid-to-liquid ratio for extracting *M. oleifera* leaves protein was determined to equal 1:8.

#### 3.1.4 Effect of the ammonium sulfate saturation level on *M. oleifera* leaves protein extraction

The effect of ammonium sulfate saturation on the extraction rate of *M. oleifera* leaves at a solid-to-liquid ratio of 1:8 with an extraction time of 6 h is shown in Figure 1D. An increase in the ammonium sulfate saturation level from 40 to 60% raised the extraction yield of *M. oleifera* leaves protein from 10.03 to 14.37 mg/g, which indicated that *M. oleifera* leaves protein mainly consisted of salt-soluble proteins. However, further improve in the saturation level of ammonium sulfate increased to 80% decreased the extraction rate of *M. oleifera* leaves protein by 0.28 times compared with that obtained with an ammonium sulfate saturation level of 60%; this finding may be due to the salt-out of protein in a solution containing a high salt concentration, which would reduce the extraction rate of *M. oleifera* leaves protein (17). Therefore, the optimal ammonium sulfate saturation level for *M. oleifera* leaves protein extraction was determined to equal 60%.

### 3.2 Results from the orthogonal experiment of the *M. oleifera* leaves protein extraction process

Based on the results of the single-factor tests, the *M. oleifera* leaves protein extraction process was optimized, and a visual analysis of the orthogonal test (Table 2) was obtained. As shown in Table 2, the primary and secondary relationship of factors affecting the extraction rate of *M. oleifera* leaves protein identified in the orthogonal experimental design was the following: B > A > C, that is, solid–liquid ratio > extraction time > ammonium sulfate saturation. The optimal extraction process combination was thus as follows: an extraction time of 6 h, a solid-to-liquid ratio of 1:8, and an ammonium sulfate saturation level of 60%.

**Table 2:** Visual analysis of the orthogonal experiment for *M. oleifera* leaves protein extraction process optimization.

	**A**	**B**	**C**	
**Number**.	**Extraction time/h**	**Solid-to-liquid ratio/g:mL**	**Ammonium sulfate/%**	**Protein extraction rate (mg.g** ^−1^ **)**
1	3	3	1	11.58
2	1	2	3	16.13
3	3	1	3	1.87
4	1	3	2	2.98
5	2	3	3	8.72
6	3	2	2	11.27
7	2	2	1	6.32
8	2	1	2	12.04
9	1	1	1	14.19
K1	31.30	24.11	30.09	
K2	25.08	33.73	24.29	
K3	24.73	23.28	26.72	
k1	10.43	8.04	10.03	
k2	8.36	11.24	8.10	
k3	8.24	7.76	8.91	
R	2.19	3.48	1.93	

### 3.3 SDS–PAGE analysis of *M. oleifera* leaves protein

**Figure 2:**
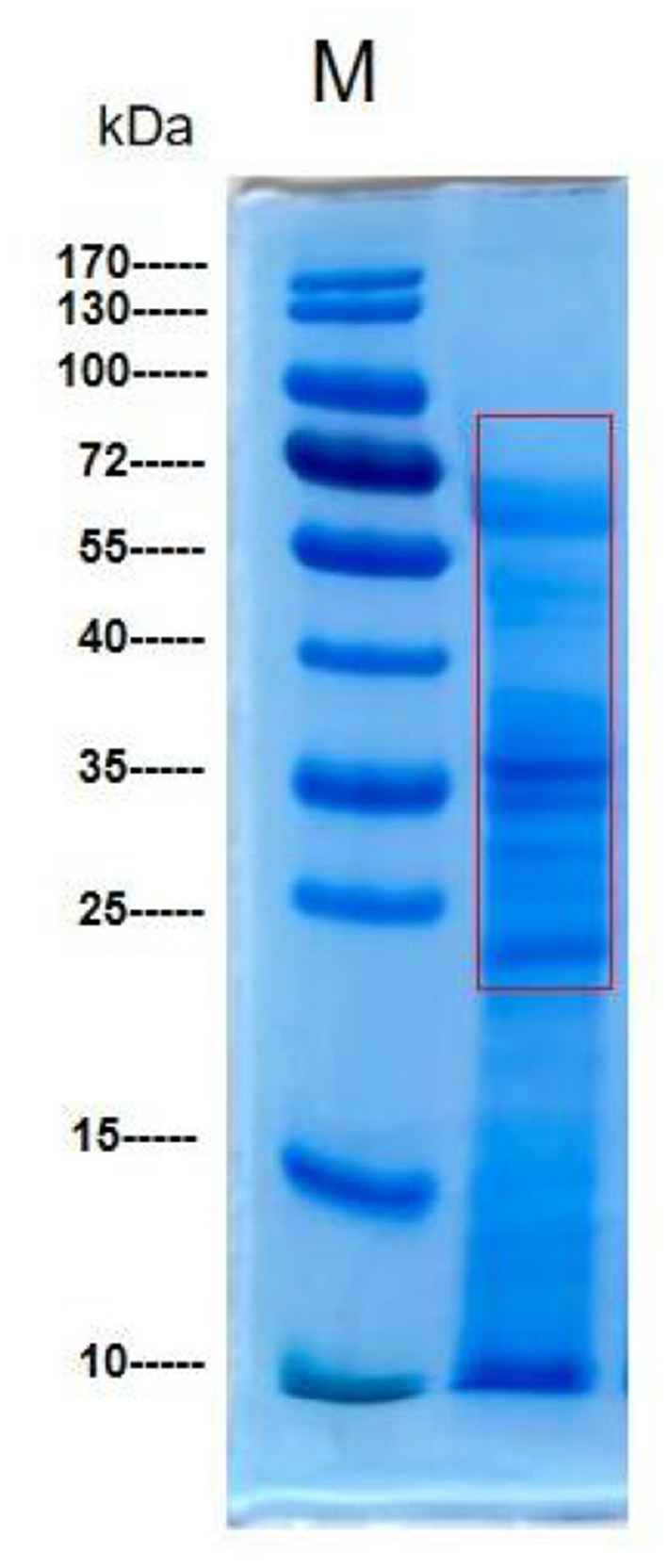
SDS–PAGE of *M. oleifera* leaves protein.

The protein bands of *M. oleifera* leaves obtained using the ammonium sulfate precipitation method were analyzed by SDS–PAGE (23). As shown in Figure 2, the protein bands were mainly concentrated at ~23 to 70 kDa, and the rest had a molecular weight of approximately 10 kDa; among all the observed bands, the highest abundance was observed for that at 35 kDa.

### 3.4 Hemagglutination titer of *M. oleifera* leaves protein

**Figure 3:**
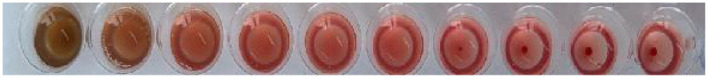
Effect of rabbit hemagglutinin.

The comparison between the agglutination activity of *M. oleifera leaves* protein and the effect of rabbit erythrocyte agglutinin is shown in Figure 3 and Table 3. The results showed that crude *M. oleifera* leaves protein can agglutinate rabbit red blood cells, and the agglutination titer of *M. oleifera* leaves protein was 2^8^.

**Table 3:** Comparison of the rabbit hemagglutinin effects.

***Moringa oleifera* leaves protein**	**Agglutination titer**								
	**2** ^1^	**2** ^2^	**2** ^3^	**2** ^4^	**2** ^5^	**2** ^6^	**2** ^7^	**2** ^8^	**2** ^9^
Strength of agglutination	++++	++++	++++	++++	++++	+++	++	+	–

#### 3.4.1 Thermal stability of *M. oleifera* leaves protein

As shown in Table 4, when the temperature was below 60°C, the *M. oleifera* leaves protein was very stable, maintaining its full hemagglutination activity, and the hemagglutination titer was 2^8^. Increasing the temperature gradually decreased the agglutination activity of *M. oleifera* leaves protein until a temperature of 110°C, which resulted in almost complete loss of hemagglutination activity. These findings show that temperature has a marked effect on *M. oleifera* leaves protein. The hemagglutination activity could be maintained under cooling conditions but might be reduced or even completely lost by increasing the temperature (28, 29).

**Table 4:** Effect of temperature on the hemagglutination activity of *M. oleifera* leaves protein.

**Temperature**	**Hemagglutination titer/2** ^**n**^								
	**2** ^1^	**2** ^2^	**2** ^3^	**2** ^4^	**2** ^5^	**2** ^6^	**2** ^7^	**2** ^8^	**2** ^9^
40°C	+++	+++	+++	++	++	++	++	–	–
50°C	+++	+++	+++	++	++	++	+	+	+
60°C	+++	+++	+++	++	++	+	+	+	–
70°C	+++	+++	+++	++	+	+	+	–	–
80°C	++	++	++	+	+	–	–	–	–
90°C	+	+	++	+	+	–	–	–	–
100°C	+	+	+	+	–	–	–	–	–
110°C	–	–	–	–	–	–	–	–	–

#### 3.4.2 Sugar-inhibition/binding specificity of *M. oleifera* leaves protein

The inhibitory effects of eight sugars on *M. oleifera* leaves protein are shown in Table 5. The hemagglutination titer of α-methyl-D-glucopyranoside was 2^2^, and those of maltose-hydrate, D-mannose, D(+) anhydrous glucose and were 2^2^, 2^3^, 2^0^, and 2^4^, respectively. In addition, the hemagglutination titer of methyl-α-D-glucopyranoside was 2^3^, that of lactose was 2^2^, and that of methyl-β-D-galactoside was 2^4^. In the reactive state of hapten inhibition, the sugar with the best inhibitory effect is called the specific sugar of lectins. The results showed that D(+) anhydrous glucose was the specific inhibitory sugar of *M. oleifera* leaves lectin because it can better inhibit the agglutination reaction of rabbit red blood cells and M. oleifera leaves protein. α-Methyl-D-glucopyranoside, lactose and maltose-hydrate also have a partial inhibitory effect on the hemagglutination reaction (30–33).

**Table 5:** Effect of sugars on the hemagglutination activity of *M. oleifera* leaves protein.

**Types of sugar**	**Hemagglutination titer/2** ^**n**^							
	**2** ^1^	**2** ^2^	**2** ^3^	**2** ^4^	**2** ^5^	**2** ^6^	**2** ^7^	**2** ^8^
α-Methyl-D-glucopyranoside	+++	+++	–	–	–	–	–	–
Maltose-hydrate	++	++	–	–	–	–	–	–
D-mannose	+++	+++	+++	–	–	–	–	–
D(+) anhydrous glucose	–	–	–	–	–	–	–	–
D-raffinose pentahydrate	+++	+++	++	++	–	–	–	–
Methyl-α-D-glucopyranoside	+++	++	+	–	–	–	–	–
Lactose	++	+	–	–	–	–	–	–
Methyl-β-d-galactopyranoside	+++	+++	++	++	–	–	–	–

#### 3.4.3 Ion-binding properties of *M. oleifera* leaves protein

Ions exert an important effect on protease activity and thermal stability (34, 35, 39). Most legume lectins are ions -binding proteins or glycoproteins. Ions stabilize the structure and increase the resistance of lectins to heat and thus inactivate proteolytic enzymes (40). The effect of ions on the hemagglutination activity of *M. oleifera* leaves protein was determined, and the results are shown in Table 6. As revealed by the results, *M. oleifera* lectin is aion-dependent lectin. K^+^, Na^+^, Mg^2+^, and NH^4+^ induced loss of the hemagglutination activity of *M. oleifera*, whereas *M. oleifera* leaves protein was found to be dependent on Ca^2+^, Fe^3+^, Fe^2+^, and Al^3+^. After Ca^2+^, Fe^3+^, Fe^2+^, and Al^3+^ treatment, the protein continued to exhibit high hemagglutination activity, and its hemagglutination titer was 2^5^-2^7^.

**Table 6:** Effects of ions on the hemagglutination activity of *M. oleifera* leaves protein.

**Salt**	**Hemagglutination titer/2** ^**n**^								
	**2** ^1^	**2** ^2^	**2** ^3^	**2** ^4^	**2** ^5^	**2** ^6^	**2** ^7^	**2** ^8^	**2** ^9^
KCl	–	–	–	–	–	–	–	–	–
AlCl_3_	+++	+++	++	++	++	+	–	–	–
NaCl	–	–	–	–	–	–	–	–	–
CaCl_2_	++	++	++	+	+	–	–	–	–
MgCl_2_	–	–	–	–	–	–	–	–	–
NH_4_Cl	–	–	–	–	–	–	–	–	–
FeCl_2_	+++	+++	++	++	++	+	–	–	–
BaCl_2_	++	+	–	–	–	–	–	–	–
FeCl_3_	+++	+++	+++	+++	+++	++	+	–	–

#### 3.4.4 Acid/base stability of *M. oleifera* leaves protein

Lectins are generally more stable to acidic and basic conditions (41). Different pH values have obvious effects on the hemagglutination activity of *M. oleifera* leaves protein. The experimental results are shown in Table 7. The highest hemagglutination activity of *M. oleifera* leaves protein was observed at pH 3.7, and the hemagglutination activity of *M. oleifera* leaves protein was completely lost at a pH value of 11.9. The reasons for this finding are as follows: at near-neutral pH, the calm electric repulsion energy is smaller than the energy of other stable protein interactions, and the *M. oleifera* leaves protein is thus stable (42, 43). However, the strong molecular electrostatic repulsion caused by the high electrostatic charge at extreme pH leads to the swelling and expansion of *M. oleifera* leaves protein molecules and the ionization of carboxyl, phenolic hydroxyl and sulfhydryl groups partially buried in the protein molecules (43, 44). These ionic groups expose themselves to the water environment, resulting in the dispersion of polypeptide chains, which fundamentally changes the spatial structure of *M. oleifera* leaves protein and thus leads to irreversible denaturation and loss of vitality.

**Table 7:** Effect of the pH value on the hemagglutination activity of *M. oleifera* leaves protein.

**pH**	**Hemagglutination titer/2** ^**n**^								
	**2** ^1^	**2** ^2^	**2** ^3^	**2** ^4^	**2** ^5^	**2** ^6^	**2** ^7^	**2** ^8^	**2** ^9^
3.7	+++	+++	+++	+++	+++	++	+	+	–
5.8	+++	+++	++	++	+	+	–	–	–
6.2	+++	++	++	++	+	–	–	–	–
7.8	++	++	+	+	–	–	–	–	–
8.3	++	+	–	–	–	–	–	–	–
9.1	++	+	–	–	–	–	–	–	–
9.5	+	+	–	–	–	–	–	–	–
10.8	–	–	–	–	–	–	–	–	–
11.2	–	–	–	–	–	–	–	–	–
11.9	–	–	–	–	–	–	–	–	–
12.2	–	–	–	–	–	–	–	–	–
13.0	–	–	–	–	–	–	–	–	–

### 3.5 Determination of the hemolytic activity of *M. oleifera* leaves protein

#### 3.5.1 Effect of *M. oleifera* leaves protein at different concentrations on hemolytic activity

**Figure 4:**
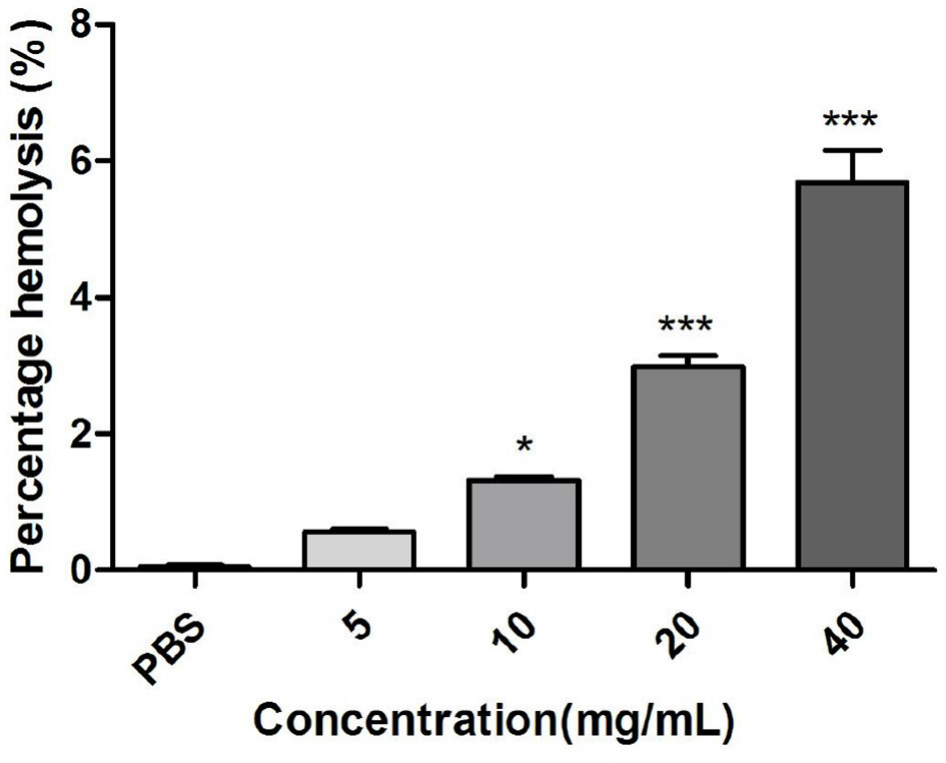
Effects of different concentrations of *M. oleifera* leaves protein on hemolytic activity. The data were compared with those of the blank control group (PBS), and the significance of the differences between groups is indicated as follows *P < 0.05 and ***P < 0.001.

The erythrocyte is considered a simple mimic of the endosome membrane and is thus a model for the study of protein-membrane interactions and for the *in vitro* assessment of the biocompatibility of important biotherapies (36). Intravenous therapeutic agents can trigger hemolysis, which is characterized by the release of hemoglobin into plasma as a result of the destruction, interference, or breakdown of red cells, which makes the search for specific proteins as carrier molecules imperative (37, 38). As shown in Figure 4, the cytocompatibility and endolytic activity of *M. oleifera* leaves proteins on rabbit red blood cells were studied using the hemolysis test, and the percentage of hemolysis was found to increase in a concentration-dependent manner. The hemolysis rate of *M. oleifera* protein at a concentration of 20 mg/mL was 5.68%, whereas that of the control group was 0.1% (*P* < 0.001).

#### 3.5.2 Effect of sugars on the hemolytic activity of *M. oleifera* leaves protein

**Figure 5:**
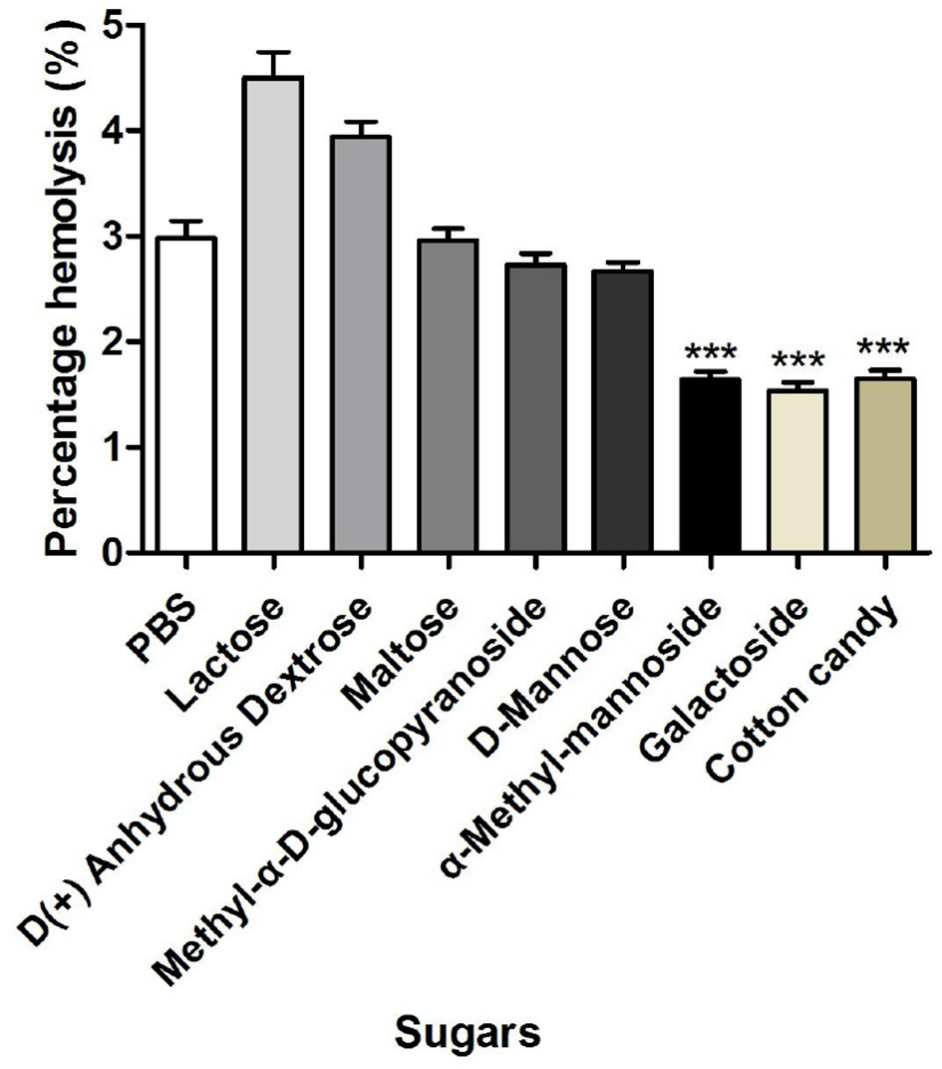
Effects of different sugars on the hemolytic activity of *M. oleifera* leaves protein. The data were compared with those of the blank control group (PBS), and the significance of the differences between groups is indicated as follows: ***P < 0.001.

As shown in Figure 5, a significant difference in the hemolysis rate was found between *M*. leaves-treated erythrocytes and erythrocytes preincubated with α-methyl-mannoside, galactoside, and raffinose (*P* < 0.001). The strong inhibitory activity of lactose may indicate that the lectin possesses an extended binding site. Furthermore, the decreased hemolytic activity of *M. oleifera* leaves protein in the presence of α-methyl-mannoside, galactoside, and raffinose further confirmed the previously demonstrated fact that *M. oleifera* leaves lectins have an affinity for ketose sugars, which inhibits the hemolytic activity of *M. oleifera* leaves protein. To improve the efficiency and reduce the side effects of specific therapeutic agents, protein-related targeted drug delivery systems have become a favorable strategy (18, 19).

#### 3.5.3 Effect of ions on the hemolytic activity of *M. oleifera* leaves protein

**Figure 6:**
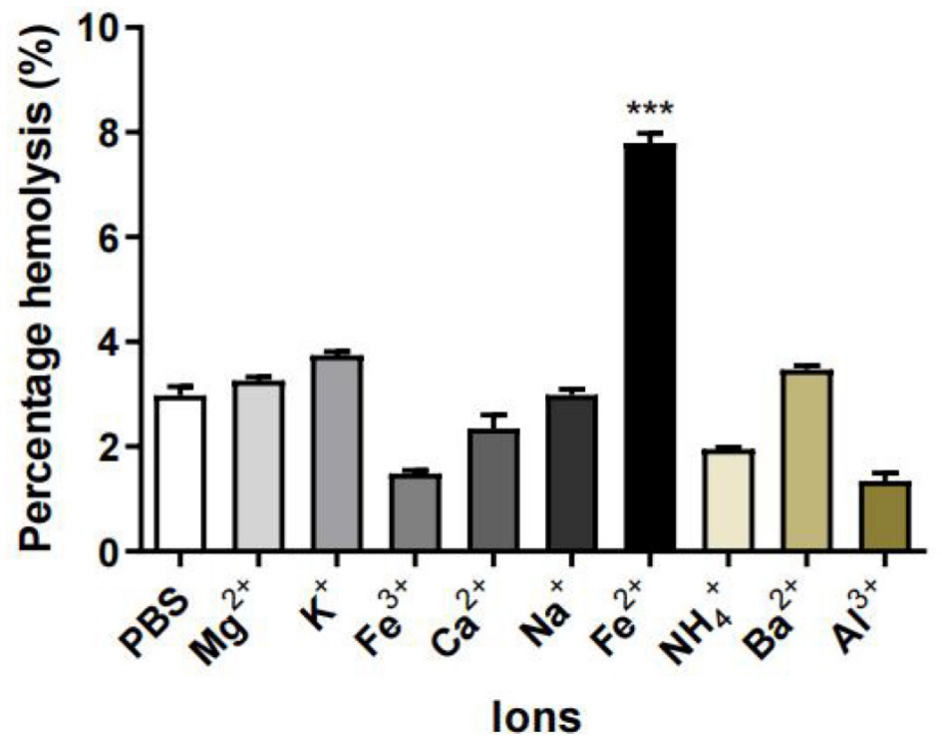
Effects of ions on the hemolytic activity of *M. oleifera* leaves protein. The data were compared with those of the blank control group (PBS), and the significance of the differences between groups is indicated as follows: ***P < 0.001.

As shown in Figure 6, among K^+^, Na^+^, Mg^2+^, NH^4+^, Fe^2+^, Ca^2+^, Fe^3+^, and Al^3+^ ions, the higher hemolysis rate was observed with erythrocytes treated with Fe^2+^ and *M. oleifera* leaves protein, indicating that Fe^2+^ caused erythrocytes to be destroyed, interfered or decomposed and released into plasma. However, Al^3+^ inhibited the hemolytic activity of *M. oleifera* protein, and similar results were reported for calcium-dependent hemolysin purified from *Cucumis acanthopanthus* (20) and *Granella anadarensis* (21).

## 4 Discussion and conclusion

The total amount of amino acids in *M. oleifera* leaves reaches 20.49%, and these leaves contain right types of essential amino acids that the human body cannot synthesize by itself or produces at a synthesis rate that cannot meet the body's needs (45–47). The most common methods for protein extraction include ultrafiltration, alkali-soluble acid precipitation, heating, and salting out, and the most traditional method is alkali-soluble acid precipitation (22, 48, 49). Based on the analysis of several extraction methods (50, 51), salting out method is the most suitable method for *M. oleifera* leaves protein extraction. The salting out method is used to separate and purify plant leaves proteins by using the characteristics of protein precipitation in a solution with a certain salt concentration (52). This method has the advantages of simple operation, low cost, and high leaves protein activity. Chen et al. (31) found that the salting out method was the most suitable method for extracting protein from leaves of *Wedelia sinensis* due to its high extraction efficiency, simple operation, good protein activity, and suitability for laboratory extraction and industrial production (53, 54). Therefore, in this study, fresh *M. oleifera* leaves were used as raw materials for the extraction of protein by the salting out method, and the effects of the extraction time, ammonium sulfate saturation and solid-to-liquid ratio on the protein extraction rate were studied to determine the best process conditions and thus lay a theoretical foundation for the deep processing of *M. oleifera* leaves protein products and promote the healthy development of the *M. oleifera* industry.

Most researchers believe that protein in food is the main cause of food allergy (24–26). Studies have found that plant-derived food allergens such as peanut, soybean, and wheat contain lectins, and these lectins may be inextricably related to allergy (55, 56). In this study, it was speculated that *M. oleifera* leaves lectin or leaves protein is the main protein responsible for the induction of allergy, and studying its basic characteristics is the first step for understanding the nature of the lectin. This study investigated various properties of *M. oleifera* lectin, particularly thermal stability, acid–base stability, ion-binding characteristics, sugar-binding specificity and hemolytic activity, to provide a theoretical basis for the development and utilization of *M. oleifera* lectin and agglutination active substances, which will pave the way for further understanding of protein anti-nutrients in *M. oleifera* lectins derived from different organisms or different types of lectins differ in their physical and chemical properties, and their biological characteristics are the basis for the wide application of lectins in different industries (57). Among them, sugar specificity is the most basic property of lectins. This property determines the hemocyte binding specificity, mitogenesis, cell recognition and agglutination properties of lectins. For legume lectins, the mutual recognition mechanism between rhizobia and host plants is also based on sugar specificity (58). Lectins are expected to become widely used in biology, biomedicine and gene engineering of disease resistance.

This study performed a preliminary exploration of the allergy-related activity of *M. oleifera* leaves protein. It was only demonstrated that the *M. oleifera* leaves protein has lectin related activity. We also identified the sequences of *M. oleifera* allergens. We took the three proteins and predicted that the sequences we identified are not homologous to the mO1 and mO2 proteins, but the 36 kd we identified is a homologous protein to Fructose 1,6 bisphosphate aldolase, FBA, and the substrate of FBA is a sugar, which has a sugar-binding sequence. According to the methodology of Nguyen et al. (59) and Maurer-Stroh et al. (60), we took the three previous sequences and used AllerCatpro to predict and study the allergenic potential of the proteins. Here we used AllerCatpro to predict that we can prove that the allergen is minimal, but our previous Western Blot experiment done on mouse serum and human serum bound to *M. oleifera* leaves protein, the bands are obvious, which is a strong evidence. But at the moment this relevant result is not published.

In the future, the purifi-cation of *M. oleifera* leaves protein allergens, the elimination of *M. oleifera* leaves protein allergens and the treatment of allergic diseases sensitized by *M. oleifera* leaves protein will be studied.

## Data Availability

The original contributions presented in the study are included in the article/supplementary material, further inquiries can be directed to the corresponding authors.
